# Intermolecular steric inhibition of Ephexin4 is relieved by Elmo1

**DOI:** 10.1038/s41598-017-04810-6

**Published:** 2017-06-30

**Authors:** Kwanhyeong Kim, Juyeon Lee, Sang-Ah Lee, Hyunji Moon, Boyeon Park, Deokhwan Kim, Young-Eun Joo, Daeho Park

**Affiliations:** 1 School of Life Sciences and Aging Research Institute, Gwangju Institute of Science and Technology, Gwangju, 61005 Korea; 2 Research Center for Cellular Homeostasis, Ewha Womans University, Seoul, 03760 Korea; 3 Department of Internal Medicine, Chonnam National University Medical School, Gwangju, 61469 Korea; 4 Department of Biomedical Science and Engineering, Gwangju Institute of Science and Technology, Gwangju, 61005 Korea

## Abstract

Ephexin4, a guanine nucleotide-exchange factor for RhoG, promotes engulfment of apoptotic cells and cancer cell migration in a RhoG-dependent manner, which is synergistically augmented by Elmo1, an Ephexin4-interacting protein. However, the underlying molecular mechanism remains elusive. Here, we report a mechanism by which Elmo1 cooperates with Ephexin4 to activate RhoG. We found that Ephexin4 activity was increased by elimination of its SH3 domain which intermolecularly interacts with the N20 region of Ephexin4. This interaction prevented RhoG from binding to Ephexin4 and thus inhibited RhoG activation. Moreover, we also found that Elmo1 associated with the SH3 domain as well as the N20 region and competed with the SH3 domain for binding to the N20 region, interrupting the interaction of the SH3 domain with the N20 region and thereby promoting RhoG binding to Ephexin4. In addition, the activity of Ephexin4 lacking the SH3 domain was comparable to that of Ephexin4 with Elmo1. Taken together, the data suggest that Elmo1 relieves the steric hindrance of Ephexin4 generated by the intermolecular interaction of the SH3 domain and makes Ephexin4 more accessible to RhoG.

## Introduction

Rho-family GTPases, a main branch of the Ras superfamily of small GTPases, cycle between GDP- and GTP-bound states to regulate cellular processes such as cell migration, phagocytosis, cellular morphogenesis, and cell growth and survival. The nucleotide-binding states of Rho-family proteins are primarily controlled by two classes of regulatory proteins, GTPase-activating proteins (GAPs) and guanine nucleotide-exchange factors (GEFs). GEFs catalyze the exchange of GDP for GTP, yielding the GTP-bound states and activating the GTPase, whereas GAPs accelerate intrinsic GTPase activity of Rho-family proteins, generating the GDP-bound inactive states^1–5^.

The 69 distinct RhoGEFs are structurally well conserved, containing a Dbl homology (DH) domain and a pleckstrin homology (PH) domain C-terminal to the DH domain. In addition to the DH–PH domain, RhoGEFs contain other protein domains involved in unique cellular functions. DH domains are responsible for catalyzing the exchange of GDP for GTP within GTPases, whereas PH domains cooperate to facilitate the activation of Rho GTPases^2, 3, 6, 7^.

The activities of most RhoGEFs are primarily regulated by interactions between their PH domain and phosphoinositides, but they can also be regulated by other mechanisms including cellular localization, phosphorylation, oligomerization, and protein–protein interactions^3–6^. In particular, the Src homology 3 (SH3) domain may modulate the activity of RhoGEFs that possess it via intra- or intermolecular autoinhibition or protein–protein interactions. For instance, the activity of Dock1, Ost or Asef is inhibited by its SH3 domain, whereas the first SH3 domain of Trio is necessary for neurite outgrowth^8–11^. Approximately one-third of RhoGEFs contain at least one SH3 domain, and RhoGEFs can be grouped into three classes based on the number and arrangement of the SH3 domain: the SH3 domain is located N-terminally to the DH–PH domain in group I GEFs and C-terminally in group II GEFs, whereas group III GEFs contain multiple SH3 domains^9^. Ephexins are a subfamily of group II RhoGEFs that directly interact with EphA receptors. To date, five members of the Ephexin family have been identified. Ephexin1 regulates axon guidance and spine morphogenesis through interaction with EphA4 and activation of RhoA^12–14^. However, the biological functions of other Ephexins are not well characterized, although it is known that Ephexin2, 3, and 5 can also activate RhoA^15–17^. In contrast to the other family members, Ephexin4 interestingly functions as a GEF for RhoG, whose activation promotes breast cancer cell migration and phagocytosis of apoptotic cells and prevents anoikis. Recently, it is reported that Ephexin4 biochemically interacts with Elmo1, which synergistically augments Ephexin4-mediated processes such as removal of apoptotic cells^18–21^.

Engulfment and cell motility protein (Elmo) is a mammalian homolog of Ced-12 that is evolutionarily conserved from worm to human. Elmo does not have intrinsic catalytic activity, but it can modulate the activity of interacting proteins or function as a scaffold protein to improve the efficiency of signal transduction. Thus, it also participates in various cellular processes such as cell migration, phagocytosis of apoptotic cells, neurite outgrowth, and myoblast fusion^22–28^. For example, via an interaction with Dock1, Elmo1 serves as a component of a bipartite GEF for Rac1. The C-terminus of Elmo1 binds to the N-terminal SH3 domain of Dock1, promoting synergistic Rac1 activation by helping Dock1 stabilize Rac1 in a nucleotide-free transition state and relieving autoinhibition caused by the SH3 domain of Dock1^8, 29, 30^.

Previously, we showed that Ephexin4 interacts with Elmo1, which synergistically promotes clearance of apoptotic cells in a RhoG-dependent manner^19^. However, the molecular mechanisms by which Elmo1 cooperates with Ephexin4 to augment RhoG activation were not elucidated. Here, we report that the SH3 domain of Ephexin4 generates steric hindrance via an intermolecular interaction with its N20 region; the intermolecular interaction prevents RhoG from binding to Ephexin4. Moreover, Elmo1 can abolish the interaction by competing with the SH3 domain for binding to the N20 region and associating with the SH3 domain, making Ephexin4 more accessible to RhoG. Consequently, the activity of Ephexin4 cooperating with Elmo1 is comparable to that of an Ephexin4 mutant lacking the SH3 domain. Taken together, these data suggest that the autoinhibition of Ephexin4 by its SH3 domain is relieved by Elmo1, which results in synergistic promotion of the activity of Ephexin4 for RhoG.

## Results

### The SH3 domain of Ephexin4 negatively regulates Ephexin4 activity

**Figure 1:**
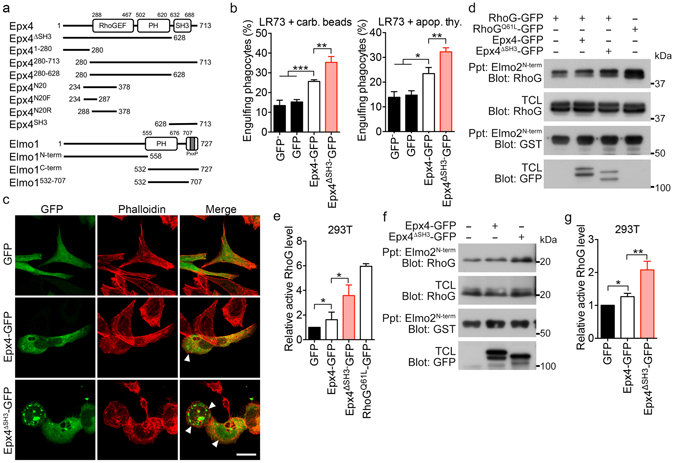
The activity of Ephexin4 is negatively regulated by its SH3 domain. (**a**) Schematic diagram of Ephexin4 and Elmo1, and their truncation mutants used in the study. Epx4, Ephexin4; GEF, Guanine nucleotide exchange factor; PH, Pleckstrin homology domain; SH3, SRC homology 3 domain. (**b**) LR73 cells were transfected with the indicated plasmids, incubated with 2 µm carboxylated beads (left) or apoptotic thymocytes (right) for 2 h, and analyzed by flow cytometry. GFP- and red fluorescence–positive phagocytes were considered to represent phagocytes that had engulfed targets. GFP^−^ indicates the non-transfected population of the cells. (**c**) LR73 cells were transfected with the indicated plasmids and stained with phalloidin. Images were obtained using confocal microscopy. Arrow heads indicate membrane ruffles. Scale bar, 20 µm. (**d**,**e**) 293 T cells were transfected with the indicated plasmids and lysed. The resultant lysates were incubated with GST-Elmo2^N-term^-bound GST beads for 1 h. Exogenous active RhoG levels were detected with immunoblotting (**d**) and quantified from four independent experiments (**e**). (**f**,**g**) 293 T cells were transfected with either Ephexin4 or Ephexin4^ΔSH3^. Endogenous active RhoG was co-precipitated with GST-Elmo2^N-term^ and detected by immunoblotting (**f**). Three independent experiments were performed, and the levels of active RhoG were quantitated (**g**). Data are shown as the mean ± standard deviation and are representative of at least three independent experiments. *P < 0.05, **P < 0.01. Immunoblots are cropped for conciseness and their full-length blots are shown in Supplementary S3. Epx4, Ephexin4; TCL, total cell lysates.

Ephexin4, which contains a long N-terminal region, followed by the tandem DH–PH domain and a C-terminal SH3 domain, is a member of group II RhoGEFs. Because the activities of RhoGEFs are often regulated by regions outside the DH–PH domain, especially by SH3 domains^2, 3, 7^, we investigated whether the C-terminal SH3 domain of Ephexin4 affected its activity. For this purpose, we generated an Ephexin4 mutant lacking the SH3 domain (Ephxin4^ΔSH3^) (Fig. 1a). First, we measured the effects of Ephexin4 or Ephexin4^ΔSH3^ on phagocytosis of apoptotic cells or carboxylate-modified beads mimicking apoptotic cells. In comparison with the GFP control or the non-transfected cells (GFP^−^), wild-type Ephexin4 promoted phagocytosis of apoptotic cells and the beads, as reported previously^19^. Interestingly, phagocytosis of either apoptotic cells or the beads induced by Ephexin4^ΔSH3^ was more extensive than that mediated by wild-type Ephexin4 (Fig. 1b). Second, because Ephexin4 also facilitates membrane ruffle formation^18^, we compared the ruffles induced by Ephexin4 and Ephexin4^ΔSH3^. Although wild type Ephexin4 expression resulted in increased membrane ruffles compared to the GFP control, the SH3 deletion mutant induced more membrane ruffles per cell and ruffles in a larger proportion of cells than wild-type Ephexin4 (Fig. 1c). Intriguingly, Ephexin4^ΔSH3^-GFP expression but not Ephexin4-GFP formed puncta-like structures, but it did not localize the subcellular compartments such as endosomes or lysosomes (Supplementary S1). These data indicate that the SH3 domain of Ephexin4 negatively affects Ephexin4-mediated processes.

Next, because Ephexin4 functions as a GEF for RhoG, we investigated whether the prominent increases in phagocytosis of apoptotic cells and membrane ruffle formation in cells expressing Ephexin4^ΔSH3^ were due to elevated RhoG activation. To measure active RhoG levels in cells, we employed a pulldown assay using Elmo2^N-term^, which interacts with GTP-loaded RhoG but not the GDP-loaded protein^25^. In this assay, Ephexin4^ΔSH3^ activated RhoG more efficiently than Ephexin4 (Fig. 1d). In comparison with control cells, active RhoG increased 3.5-fold in cells expressing Ephexin4^ΔSH3^, but only 1.7-fold in cells expressing wild-type Ephexin4 (Fig. 1e). In addition, prominently increased active RhoG by Ephexin4^ΔSH3^ was also observed at endogenous levels (Fig. 1f). Ephexin4^ΔSH3^ increased the levels of active RhoG 2-fold, whereas wild-type Ephexin4 increased the levels of active RhoG 1.3-fold (Fig. 1g). Taken together, these data suggest that the SH3 domain of Ephexin4 functions as an inhibitory domain to suppress RhoG activation.

### The SH3 domain of Ephexin4 mediates homotypic interaction

**Figure 2:**
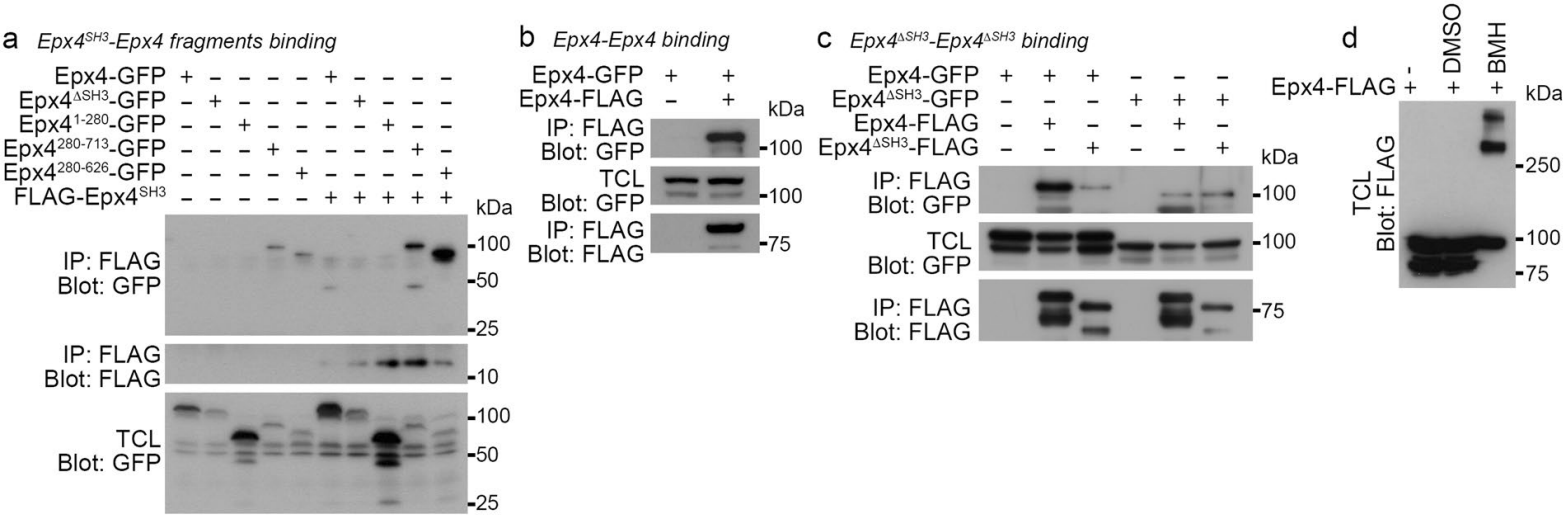
The SH3 domain mediates homotypic interaction of Ephexin4. (**a**) 293 T cells were transfected with the indicated plasmids. Two days after transfection, the SH3 domain of Ephexin4 was immunoprecipitated with anti-FLAG antibody and the co-immunoprecipitated proteins were detected with anti-GFP antibody. (**b**) GFP- and FLAG-tagged Ephexin4 were expressed in 293 T cells and then lysed. Ephexin4-FLAG was immunoprecipitated with anti-FLAG antibody, and co-immunoprecipitated proteins were detected with anti-GFP antibody. (**c**) 293 T cell were transfected with the indicated plasmids, lysed and immumoprecipitated with anti-FLAG antibody. Co-immunoprecipitated proteins were detected with anti-GFP antibody. (**d**) 293 T cells were transfected with Ephexin4-FLAG. Two days after transfection, the cells were incubated in the presence or absence of 0.25 mM BMH at room temperature for 1 h. Ephexin4-FLAG was detected by immunoblotting with anti-FLAG antibody. Immunoblots are cropped for conciseness and their full-length blots are shown in Supplementary S4. Epx4, Ephexin4; TCL, total cell lysates.

Next, we investigated the mechanism by which the SH3 domain repressed the activity of Ephexin4. Previous evidence indicates that SH3 domains in various RhoGEFs or unconventional GEFs often regulate the activation of the GEFs via mediating protein-protein interaction with themselves^8–11^. Thus, it is possible that a similar mechanism could be applied to Ephexin4, that is, the SH3 domain of Ephexin4 interacts with itself and exerts autoinhibition. To test this possibility, we generated various truncation mutants of Ephexin4 (Fig. 1a) and evaluated their interaction with the SH3 domain of Ephexin4. Among the mutants, Ephexin4^280-626^, containing the tandem DH–PH domain of Ephexin4, exhibited the strongest association with the SH3 domain (Fig. 2a), suggesting that the SH3 domain intra- or intermolecularly interacts.

In order to test whether the interaction occurs intra- or intermolecularly, we first tested whether Ephexin4 associated with itself using differently tagged Ephexin4s. FLAG-tagged Ephexin4 was co-immunoprecipitated with GFP-tagged Ephexin4 (Fig. 2b), which indicates that Ephexin4 engages in a homotypic intermolecular interaction. In order to evaluate whether the homotypic interaction was mediated by the SH3 domain, the association between full length Ephexin4 and Ephexin4^ΔSH3^ was evaluated. Interestingly, although full-length Ephexin4 showed robust interaction with full-length Ephexin4, Ephexin4^ΔSH3^ minimally associated with either full-length Ephexin4 or Ephexin4^ΔSH3^ (Fig. 2c), indicating that the SH3 domain is necessary for Ephexin4-Ephexin4 interaction. Additionally, we tested how Ephexin4 was oligomerized. When Ephexin4 was crosslinked between its sulfhydryl groups of cysteine residues by bis(maleimido)hexane (BMH), oligomerized Ephexin4 was mainly observed above 250 kDa which is 3-fold higher than that of Ephexin4 normally detected (Fig. 2d). Taken together, these data suggest that Ephexin4 could form a trimer through the homotypic intermolecular association, which is mediated by the SH3 domain.

### The N20 region of Ephexin4, required for Elmo1 binding, interacts with the SH3 domain

**Figure 3:**
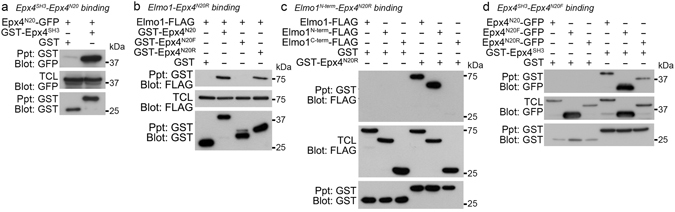
The SH3 domain associates with the N20 region of Ephexin4. (**a**–**d**) 293 T cells transfected with the indicated plasmids were lysed, and then the lysates were incubated and precipitated with glutathione–sepharose beads. Co-precipitated proteins were detected with anti-GFP (**a**,**d**) or anti-FLAG antibody (**b**,**c**). Immunoblots are cropped for conciseness and their full-length blots are shown in Supplementary S5. Epx4, Ephexin4; TCL, total cell lysates.

The interaction between the SH3 domain and Ephexin4^280-626^ was reminiscent of the interaction between Elmo1 and Ephexin4 because the amino acids of Ephexin4^280-626^ contains the Elmo1-binding region of Ephexin4 (the N20 region of Ephexin4 containing amino acids 224–378, originally identified through a yeast two-hybrid screen in which Elmo1 was used as bait). Thus, we next performed pulldown assays to test whether the SH3 domain could associate with the N20 region of Ephexin4. Intriguingly, the N20 region was strongly co-precipitated with the SH3 domain (Fig. 3a). Elmo1 more specifically bound the N20R region, the shortest fragment of Ephexin4 necessary for the association with the Elmo1, but not the N20F region (Fig. 3b and c). We furthermore investigated whether the SH3 domain binding region was overlapped with the Elmo1 binding region, N20R. In contrast to the Elmo1 binding propensity, the SH3 domain associated with N20F more strongly than N20R (Fig. 3d). Collectively, these data suggest that the SH3 domain of Ephexin4 mediates the homotypic interaction through associating with the N20 region necessary for Elmo1 association, implying possible involvement of Elmo1 in the regulation of Ephexin4 by its SH3 domain.

### The SH3 domain impedes RhoG access, which is disrupted by Elmo1

**Figure 4:**
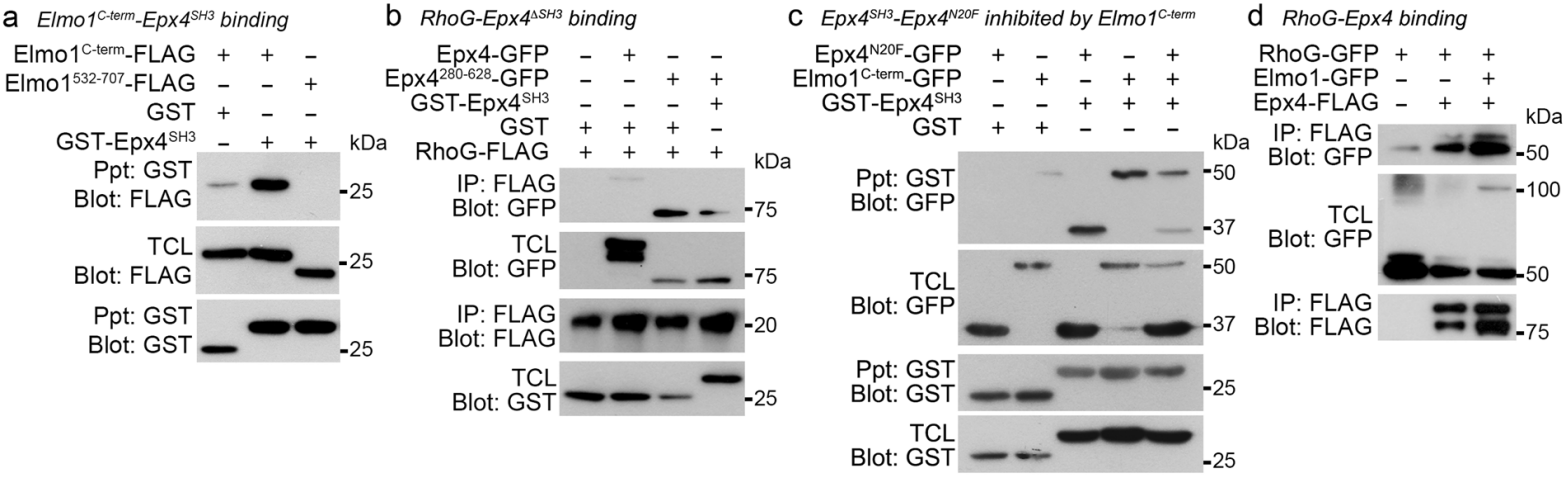
The association between the SH3 domain and the N20 region impeding RhoG access is disrupted by Elmo1. (**a**) 293 T cells were transfected with the indicated plasmids and lysed. The lysates were incubated with glutathione-sepharose beads. GST-tagged proteins were precipitated with the beads and then co-precipitated proteins were detected with anti-FLAG antibody. (**b**) 293 T cells were transfected with the indicated plasmids and lysed in lysis buffer containing 10 µM EDTA. FLAG-tagged proteins were precipitated with anti-FLAG antibody, and RhoG binding was detected with anti-GFP antibody. (**c**) 293 T cells were transfected with the indicated plasmids, and proteins that co-precipitated with the SH3 domain of Ephexin4 were detected with anti-GFP antibody. (**d**) Nucleotide-free RhoG associated with Ephexin4 was evaluated in 293 T cells in the presence or absence of exogenous Elmo1 as in (b). Immunoblots are cropped for conciseness and their full-length blots are shown in Supplementary S6. Epx4, Ephexin4; TCL, total cell lysates.

Although the association between Elmo1 and Ephexin4 is mediated by the N-terminus of Elmo1 and the N20 region of Ephexin4 (Fig. 3b and c), it is well known that the C-terminus of Elmo1 interacts with the SH3 domain of Dock1. Thus, because there was a possibility that the SH3 domain of Ephexin4 could interact with the C-terminus of Elmo1, we tested interaction between the C-terminus of Elmo1 and the SH3 domain of Ephexin4. Interestingly, the SH3 domain of Ephexin4 co-precipitated the C-terminus of Elmo1, and this was completely abrogated by the deletion of the PxxP motif located in the C-terminus of Elmo1 (Elmo1^532-707^) (Fig. 4a), indicating that the interaction between the SH3 domain and C-term Elmo1 is mediated by the proline rich region of the C-terminus of Elmo1. Taken together, the data indicate that N-term and C-term Elmo1 associates with the N20 region and the SH3 domain of Ephexin4, respectively.

Next, the intermolecular interaction of Ephexin4 mediated by the SH3 domain as well as the prominent effects of Ephexin4^ΔSH3^ on Ephexin4-induced RhoG activation, strongly suggested that the SH3 domain generates steric hindrance to constrain the activation of Ephexin4. Therefore, we next investigated whether the inhibitory effects of the SH3 domain resulted from impeding RhoG access to the DH domain by the SH3 domain. To address this, we evaluated RhoG binding to Ephexin4 in the presence or absence of the SH3 domain. Intriguingly, nucleotide-free RhoG binding to the tandem DH–PH domain of Ephexin4 (Ephexin4^280-628^) was much stronger than wild-type Ephexin4. However, this strong association of nucleotide-free RhoG with Ephexin4^280-628^ was weakened by the expression of the SH3 domain (Fig. 4b), implying that the SH3 domain behaves as an obstacle domain preventing RhoG from binding to the DH domain of Ephexin4.

Next, from a perspective of steric hindrance caused by the SH3 domain, we scrutinized a mechanism by which Ephexin4 and Elmo1 cooperatively promote RhoG activation. It is plausible that Elmo1 increases the activity of Ephexin4 through competing with the SH3 domain for binding to the N20 region because the N20 region is the common binding site for both the SH3 domain and Elmo1. Thus, Elmo1 disrupts the intermolecular interaction and relieves the steric hindrance caused by the SH3 domain. To address this possibility, we first tested whether Elmo1 could disrupt the interaction between the SH3 domain and the N20 region. Interestingly, the association between Ephexin4^N20F^ and Ephexin4^SH3^ was remarkably diminished when the C-terminus of Elmo1 was present (Fig. 4c), indicating that Elmo1 interrupts the interaction of the SH3 domain and might eliminate the steric hindrance caused by the SH3 domain.

Abolishing the interaction of the SH3 domain with the N20 region by Elmo1 could augment RhoG binding to Ephexin4 through reliving the steric hindrance. Thus, we next investigated whether the presence of Elmo1 would make Ephexin4 more accessible to RhoG. Although nucleotide-free RhoG was substantially co-precipitated with Ephexin4, the amount of co-precipitated RhoG with Ephexin4 was much higher in the presence of Elmo1 (Fig. 4d), suggesting that Elmo1 enhances the binding of RhoG to Ephexin4 through disrupting the interaction of the SH3 domain. Taken together, these data suggest that that Elmo1 relieves the steric inhibition by disrupting the interaction between the N20 region and the SH3 domain of Ephexin4, making Ephexin4 more accessible to RhoG.

### Elmo1 is necessary for activation of Ephexin4

**Figure 5:**
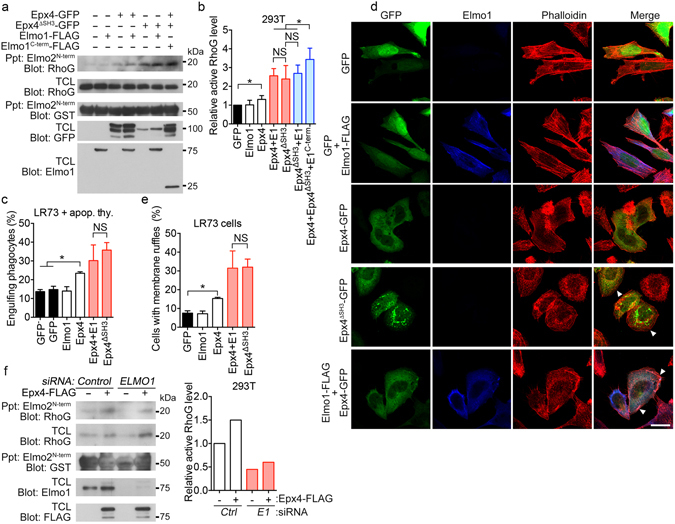
Comparison of the activity of Epheinx4^ΔSH^ with that of Ephexin4 and Elmo1. (**a**,**b**) 293 T cells were transfected with the indicated plasmids and lysed. The resultant lysates were incubated with GST-Elmo2^N-term^ binding to glutathione–Sepharose beads. Co-precipitated endogenous RhoG was detected with anti-RhoG antibody. The assay was performed 3 times. The representative blot (**a**) and quantified data (**b**) are shown. (**c**) The indicated proteins were expressed in LR73 cells, and then the cells were incubated with apoptotic thymocytes for 2 h. LR73 cells ingesting apoptotic thymocytes were analyzed by flow cytometry. (**d**,**e**) LR73 cells were transfected with the indicated plasmids and stained with Alexa Fluor 488–conjugated phalloidin and anti-Elmo1 antibody (**d**). At least three hundred GFP (and Elmo1) positive cells were analyzed from randomly selected areas of three independent experiments, and GFP (and Elmo1) positive cells with membrane ruffles were counted (**e**). Arrow heads indicate membrane ruffles. Scale bar, 20 µm. (**f**) 293 T cells were transfected with Ephexin4 and either *control* siRNA or *Elmo1* siRNA. 2 days after transfection, expression of endogenous Elmo1 and overexpressed Ephexin4 were evaluated, and active RhoG levels were detected using the GST-Elmo2^N-term^ precipitation method (left) and quantified (right). Data are shown as the mean ± standard deviation and are representative of at least three independent experiments. *P < 0.05. Immunoblots are cropped for conciseness and their full-length blots are shown in Supplementary S7. Epx4, Ephexin4; E1, Elmo1; TCL, total cell lysates.

Next, we evaluated whether the negative consequence of the SH3 domain could be removed by Elmo1. In order to test this, we first compared RhoG activation mediated by Ephexin4^ΔSH3^ to that mediated by Ephexin4 and Elmo1 together. Active RhoG levels were higher in cells expressing Ephexin4^ΔSH3^ than in cells expressing wild-type Ephexin4. In addition, the level of RhoG activated by Ephexin4^ΔSH3^ was not statistically different from that of Ephexin4 and Elmo1 although the levels of active RhoG is slightly higher in cells expressing both Ephexin4 and Elmo1. Moreover, the co-expression of Elmo1 and Ephexin4^ΔSH3^ could not increase active RhoG levels further (Fig. 5a and b), supporting the idea that Elmo1 eliminates the inhibitory effects of the SH3 domain on the GEF activity of Ephexin4. Alleviation of SH3 domain–induced inhibition by Elmo1 was further evaluated in phagocytosis assays and monitoring of membrane ruffle formation. Phagocytes expressing Ephexin4 promoted engulfment of apoptotic cells relative to the control, and the elevated efferocytosis by Ephexin4 was further increased upon expression of Elmo1. However, the level of efferocytosis mediated by Ephexin4 with Elmo1 was similar to that induced by Ephexin4^ΔSH3^ alone (Fig. 5c). Furthermore, the amount of membrane ruffles formed upon expression of Ephexin4^ΔSH3^ was comparable with that mediated by Ephexin4 with Elmo1 (Fig. 5d and e), indirectly supporting the idea that the intermolecular steric inhibition mediated by the SH3 domain is relieved by Elmo1.

**Figure 6:**
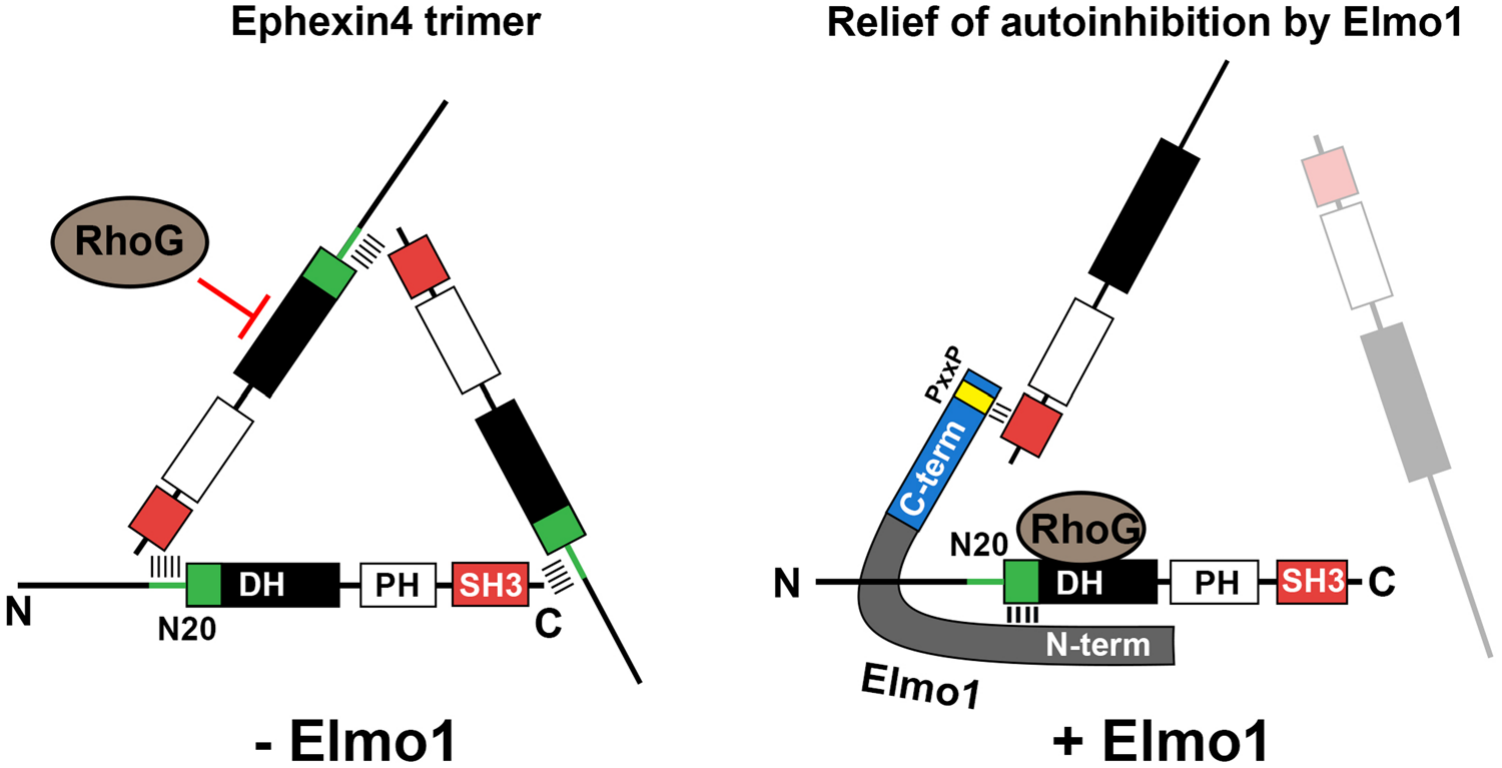
Graphical demonstration for a putative mechanism how Elmo1 relieves the intermolecular steric inhibition of Ephexin4. Intermolecular association of Ephexin4 mediated by interaction of the SH3 domain with the N20 region of Ephexin4 forms a trimeric complex, whose conformation could be a triangle shape. This conformation prevents RhoG access to the DH domain of Ephexin4. Elmo1 interferes the interaction between the SH3 domain and the N20 region of Ephexin4 and then the geometry of Ephexin4 is disrupted, which facilitates RhoG access to the DH domain of Ephexin4.

Next, we investigated whether Elmo1 is necessary for activation of Ephexin4 through depleting Elmo1 using siRNA. Ephexin4 increased the level of active RhoG in *control* siRNA transfected cells. However, the active RhoG levels prominently decreased in *Elmo1* siRNA transfected cells at the basal state, which was weakly promoted by Ephexin4 (Fig. 5f), indicating that Elmo1 is required for the activity of Ephexin4. Taken together, the data suggest that Elmo1 augments the activity of Ephexin4 by relieving the steric hindrance of Ephexin4 caused by its SH3 domain, resulting in increase of RhoG binding to Ephexin4, and thus cooperates with Ephexin4 for activation of RhoG (Fig. 6).

## Discussion

Several members of the RhoGEF family exist in an inactive state that is maintained by autoinhibition through intra- or intermolecular interactions. Intramolecular inhibition of RhoGEFs is often mediated by interaction between the DH and PH domains, or between the DH domain and regulatory regions. SH3 domains often serve as regulatory domains of RhoGEFs such as Src, Hck, and Tec family tyrosine kinases^31–36^. Most SH3 domains bind to a consensus core, Pro-X-X-Pro, with high affinity^37, 38^. Ephexin4 is a RhoGEF with a SH3 domain at the C-terminus (i.e., a group II RhoGEF). In addition, the N20 region of Ephexin4 possesses multiple PxxP motifs. Thus, it is persuasive that the SH3 domain of Ephexin4 associates with itself, which causes autoinhibition of Ephexin4. In a future study, it would be interesting to test whether the PxxP motifs that are N-terminal to the DH domain mediate the intermolecular interaction of the SH3 domain.

There are five Ephexin homologs belonging to group II of the RhoGEFs. Among them, the DH–PH and SH3 domains are highly conserved, whereas the N-terminal regions are variable. Previous work showed that Elmo also interacts with other members of Ephexin family proteins^19^. Intriguingly, Ephexin family proteins function as GEF proteins for RhoA rather than RhoG except Ephexin4. Thus, it will be interesting to investigate whether Ephexin homologs are also regulated by their SH3 domains and, if so, whether Elmo is commonly involved in relief of the inhibition.

We observed that RhoG activation by Ephexin4 and Elmo1 together was higher than that by Ephexin4^ΔSH3^ although the difference is not statistically significant. These data imply that the roles of Elmo1 for the activation of RhoG by Ephexin4 is not limited to disrupting the intermolecular interaction and relieving the steric hindrance. Elmo1 possibly plays additional roles for RhoG activation by Ephexin4. Elmo1 might cooperate with Ephexin4 to stabilize RhoG in a nucleotide-free transition state, similar to Rac1 activation by Dock1 and Elmo1, which results in the higher GEF activity of Epheinx4 with Elmo1 than that of Ephexin4 alone or Ephexin4 without the SH3 domain.

Although we found that the SH3 domain of Ephexin4 mediates the Ephexin4-Ephexin4 interaction, the interaction between Ephexin4 and Ephexin4^ΔSH3^ was also abrogated. This could be caused by stronger interaction between full-length Ephexin4 molecules than that of Ephexin4-Ephexin4^ΔSH3^. In the mixture of Ephexin4 and Ephexin4^ΔSH3^, full length Ephexin4 associate with itself because it has two binding sites each other. However, Ephexin4^ΔSH3^ has only one binding site for full-length Ephexin4 and vice versa. Thus, Ephexin4-Ephexin4 generates stronger interaction than Ephexin4-Ephexin4^ΔSH3^. In a trimeric complex of Ephexin4, the interaction is similar. Full-length Ephexin4 tends to be included in a trimeric complex but Ephexin4^ΔSH3^ is unlikely included in a trimer. This may explain that Ephexin4-Ephexin4^ΔSH3^ interaction is also abrogated.

In addition, we found that the presence of Elmo1 did not disrupt the full-length level of Ephexin4-Ephexin4 interaction (Supplementary S2). These data suggest that Elmo1 may link a Ephexin4 molecule to another Ephexin4 molecule although it abolishes interaction of the SH3 domain with the N20 region. This is plausible because Elmo1 has two binding sites for Ephexin4 and *vice versa*. C-term Elmo1 or N-term Elmo1 or both disrupt the interaction between the SH3 domain and the N20 region of Ephexin4 and simultaneously associate with the N20 region and the SH3 domain, respectively. Thus, Elmo1 bridges two Ephexin4 molecules (Fig. 6). In fact, this model explains the phenomenon that Elmo1 does not disrupt the full-length level of Ephexin4-Ephexin4 interaction.

The biochemical approaches used in this study were insufficient to determine the more detailed mechanisms by which Ephexin4 is autoinhibited by the SH3 domain and Elmo1 relieves the inhibition. The crystal structure of the Ephexin4 and Elmo1 complex would aid greatly in elucidating the mechanisms underlying the regulation of GEF activity of Ephexin4. Collectively, the data presented here suggest that the intermolecular interaction of the SH3 domain of Ephexin4 hinders RhoG access to the DH domain, resulting in autoinhibition. Binding of Elmo1 to Ephexin4 abrogates the interaction of the SH3 domain and relieves autoinhibition. Thus, Elmo1 cooperates with Ephexin4 and synergistically increases the GEF activity of Ephexin4. However, the more detailed mechanisms of Ephexin4 regulation by Elmo1 could be elucidated through crystallography in future studies. Because Ephexin family proteins are involved in neuronal cell development, cancer metastasis, and phagocytosis of apoptotic cells, the information obtained in this study will ultimately facilitate development of therapeutics for diseases, such as autoimmune diseases, that involve Ephexin family proteins.

## Methods

### Plasmids and antibodies

The Elmo and Ephexin4 constructs used in this study were described previously^19^. All truncated Ephexin4 mutants were generated by a PCR-based strategy from the Ephexin4 cDNA. The antibodies used in this study were anti-FLAG (Sigma, M2 and M5), anti-GFP (Santa Cruz Biotechnology, FL), anti-GST (Santa Cruz Biotechnology, B-14), anti-Elmo1 (Abcam, ab174298), anti-EEA1(Santa Cruz, sc-6415), anti-LAMP1(abcam, ab25630), anti-Rab7(Cell signaling, #9367) and anti-RhoG (Santa Cruz Biotechnology, 1F3 B3 E5).

### Cell culture and transfections

293 T cells, HeLa cells, and L cells were maintained in DMEM supplemented with 10% FBS and 1% penicillin/streptomycin/glutamine. LR73 cells were maintained in alpha-MEM supplemented with 10% FBS and 1% penicillin/streptomycin/glutamine. 293 T cells were transfected with calcium phosphate (Promega), and LR73 cells and L cells were transfected with Lipofectamine 2000 (Invitrogen).

### Immunoblotting and immunoprecipitation

293 T cells were transfected, washed, and then lysed with lysis buffer containing 50 mM Tris-Cl (pH 7.6), 150 mM NaCl, 10 mM NaPP, 10 mM NaF, 1 mM Na_3_VO_4_, 1% Triton X-100, and protease inhibitor cocktail. For immunoprecipitation assays, the lysates were incubated with the appropriate antibody-conjugated protein-A/G beads or glutathione–Sepharose beads for 2 h at 4 °C. After incubation, the beads were washed extensively with wash buffer containing 20 mM HEPES (pH 7.4), 180 mM NaCl, 5 mM NaF, 1 mM Na_3_VO_4_, 0.1% Triton X-100, 10% glycerol, and protease inhibitor cocktail. Finally, proteins in the lysates and precipitated on beads were assessed by immunoblotting for the corresponding tags. For RhoG binding assay, lysis buffer additionally contains 10 µM EDTA and other steps follow the immunoprecipitation assay above.

### Detection of active RhoG

293 T cells or HeLa cells were transfected with the indicated plasmids; 48 h after transfection, the cells were washed with cold PBS and lysed. To precipitate active RhoG, the lysates were incubated with GST-ELMO2^1-360^ bound to glutathione–Sepharose beads for 1 h at 4 °C. After that, the beads were extensively washed, and bound proteins were separated through SDS-PAGE and detected by immunoblotting. The amount of ELMO2^1-360^-bound RhoG was normalized to the total amount of RhoG in cell lysates for the comparison of active RhoG levels. Active RhoG levels were quantitated with ImageJ.

### Immunostaining

LR73 cells were plated on 18 mm Ø glass coverslips in a 12-well non-culture plate the day before transfection, and the cells were transfected with the indicated plasmids. One day after transfection, cells were washed with cold PBS, fixed in 4% paraformaldehyde for 15 min, and permeabilized with 0.1% Triton X-100 for 5 min. Next, permeabilized cells were blocked with 1% BSA for 30 min and stained with Alex Fluor 594–conjugated phalloidin (Life Technologies) and anti-Elmo1 antibody for 1 h at room temperature. After actin staining, nuclei were stained with Hoechst 33342 (Invitrogen), and the cells were analyzed on a Zeiss Axio Imager D2. For the study of co-localization of Ephexin4^ΔSH3^-GFP with subcellular localization markers, 7.5 × 10^4^ LR73 cells were transiently transfected pEBB-Ephexin4^ΔSH3^-GFP on the 18 mm Ø cover glasses in a 12-well plate. One day after transfection, the cells were fixed with 4% paraformaldehyde in PBS for 15 min at RT, rinsed with PBS for 2 times and incubated with 0.1% PBST to permeabilize the cells. After that, the cells were incubated with primary antibody in 3% BSA in PBS at 4 °C overnight, washed with PBS for 5 min 2 times and incubated with secondary antibody conjugated with alexa fluorchromes for 1 h at RT. Images were acquired on Olympus FV1000 SPD.

### Phagocytosis assay

LR73 cells or L cells on 24-well plates were transfected with the indicated plasmids. One day after transfection, engulfment assays were performed as follows. Transfected cells were incubated with either 2 μm carboxylate-modified red fluorescent beads (Invitrogen) or TAMRA-labeled apoptotic thymocytes in an incubator with 5% CO_2_ at 37 °C for 2 h. Next, the phagocytes were extensively washed with ice-cold PBS, trypsinized, resuspended, and analyzed by flow cytometry. Transfected cells were identified by GFP fluorescence, and engulfment targets were identified by red fluorescence. Double-positive cells were considered to represent phagocytes engulfing targets. Flow cytometry data were analyzed using the FlowJo software.

### Purification of GST-ELMO2^1-360^

BL21(DE3) cells transformed with pGEX-4T-2-Elmo2^1-360^ were induced with 1 mM IPTG for 4 h, and then lysed by sonication. The resultant lysates were incubated with glutathione–Sepharose 4B beads for 2 h at 4 °C. The beads were washed thoroughly and resuspended in washing buffer containing 20% glycerol.

### Cross-linking assay

Chemical cross-linking assay was performed based on the manufacturer’s protocol. Briefly, the method is as follows. 293 T cells were transfected with Ephexin4 and the cells were rinsed twice with PBS and harvested 2 days after transfection. The cells were resuspended in conjugation buffer (PBS pH7.3 and 5 mM EDTA), incubated with DMSO or BMH (0.25 mM, Thermo Scientific) at room temperature for 1 h, and then terminated by adding 25 mM dithiothreitol (Sigma). After that, the cells were lysed and proteins were detected by immunoblotting.

### Statistical analysis

All data are shown as means ± standard deviation. Each experiment was performed independently at least three times, and statistical significance of differences was evaluated by two-tailed t test using the GraphPad Prism 6 software. p < 0.05 was taken to indicate a significant difference.

## Electronic supplementary material

Supplementary Information
