# Supplementary material for: Intermolecular steric inhibition of Ephexin4 is relieved by Elmo1
Source: Sci Rep. 2017 Jun 30;7:4404. doi: 10.1038/s41598-017-04810-6 (PMC5493634; doi:10.1038/s41598-017-04810-6)
Supplement: Supplementary file 1 — Supplementary Information [file 41598_2017_4810_MOESM1_ESM.pdf]

Intermolecular steric inhibition of Ephexin4 is relieved by Elmo1

Kwanhyeong Kim[1,2], Juyeon Lee[1, 2], Sang-Ah Lee[1], Hyunji Moon[1,2], Boyeon Park[1], Deokhwan Kim[1], Young-Eun Joo[3], Daeho Park[1, 2, 4]

1. School of Life Sciences and Aging Research Institute, Gwangju Institute of Science and Technology, Gwangju 61005, Korea

2. Research Center for Cellular Homeostasis, Ewha Womans University, Seoul 03760, Korea

3. Department of Internal Medicine, Chonnam National University Medical School, Gwangju 61469, Korea

4. Department of Biomedical Science and Engineering, Gwangju Institute of Science and Technology, Gwangju 61005, Korea

Correspondence to

Daeho Park

School of Life Sciences

Gwangju Institute of Science and Technology

Gwangju 61005, Korea

Tel.: 82-62-715-2890

Fax: 82-62-715-2484

Email: daehopark@gist.ac.kr

Supplementary S1

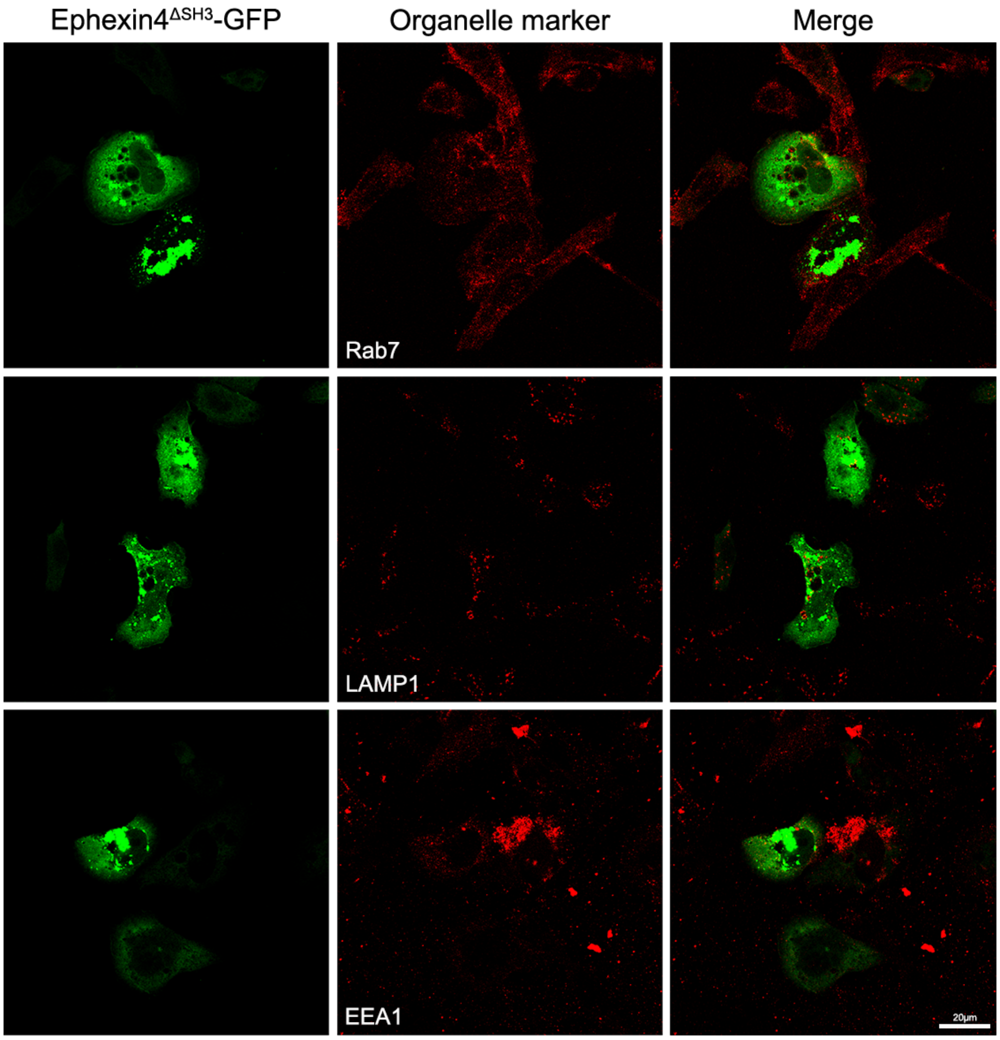

LR73 cells were transfected with Ephexin4$^{\Delta SH3}$-GFP. 1 day after transfection, the cells were permeabilized and stained with anti-Rab4, anti-LAMP1, or anti-EEA1. Images were obtained using confocal microscopy. Scale bar, 20 μm.

Supplementary S2

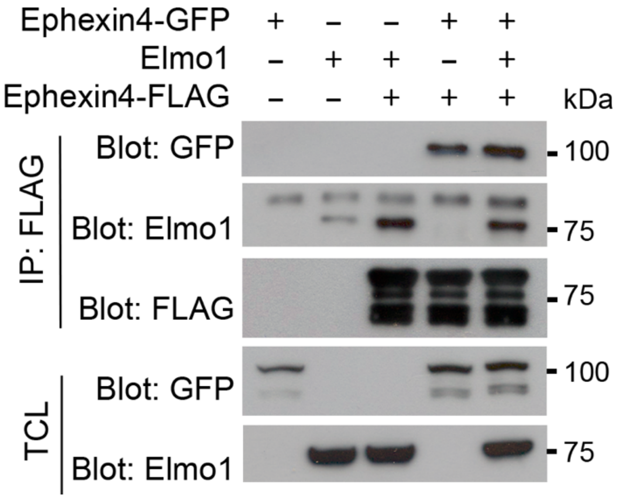

293T cells transfected with the indicated plasmids were lysed 2 day after transfection, and then the lysates were immunoprecipitated with anti-FLAG antibody. Co-immunoprecipitated proteins were detected with anti-GFP, anti-Elmo1 or anti-FLAG through western blotting. TCL, total cell lysates.

Supplementary S3

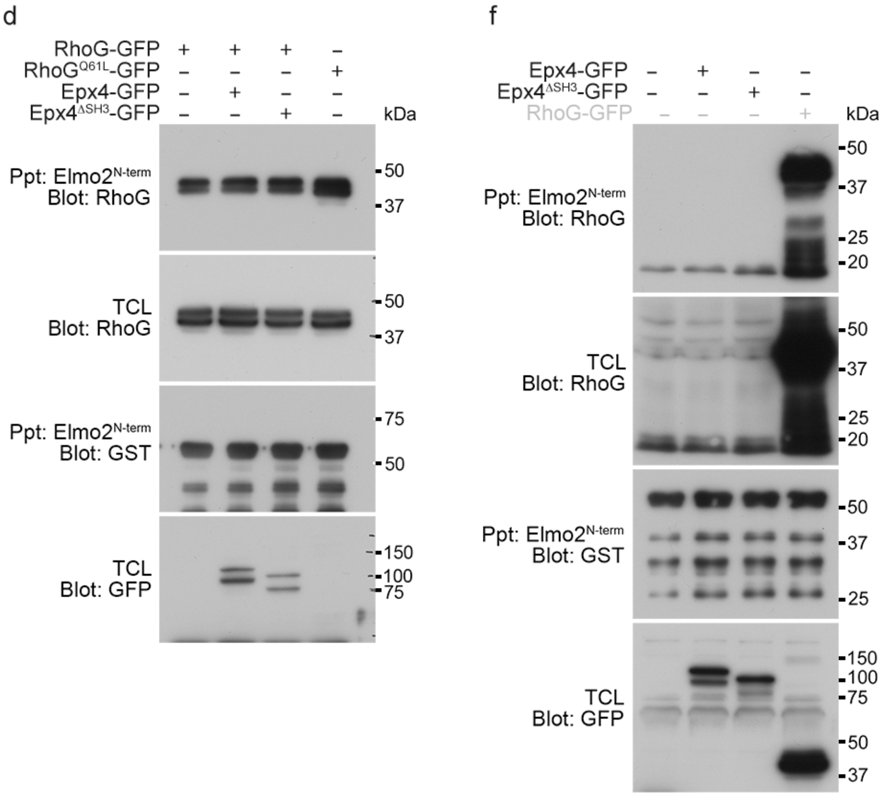

Uncropped western blots for the blots used in Figure 1

Supplementary S4

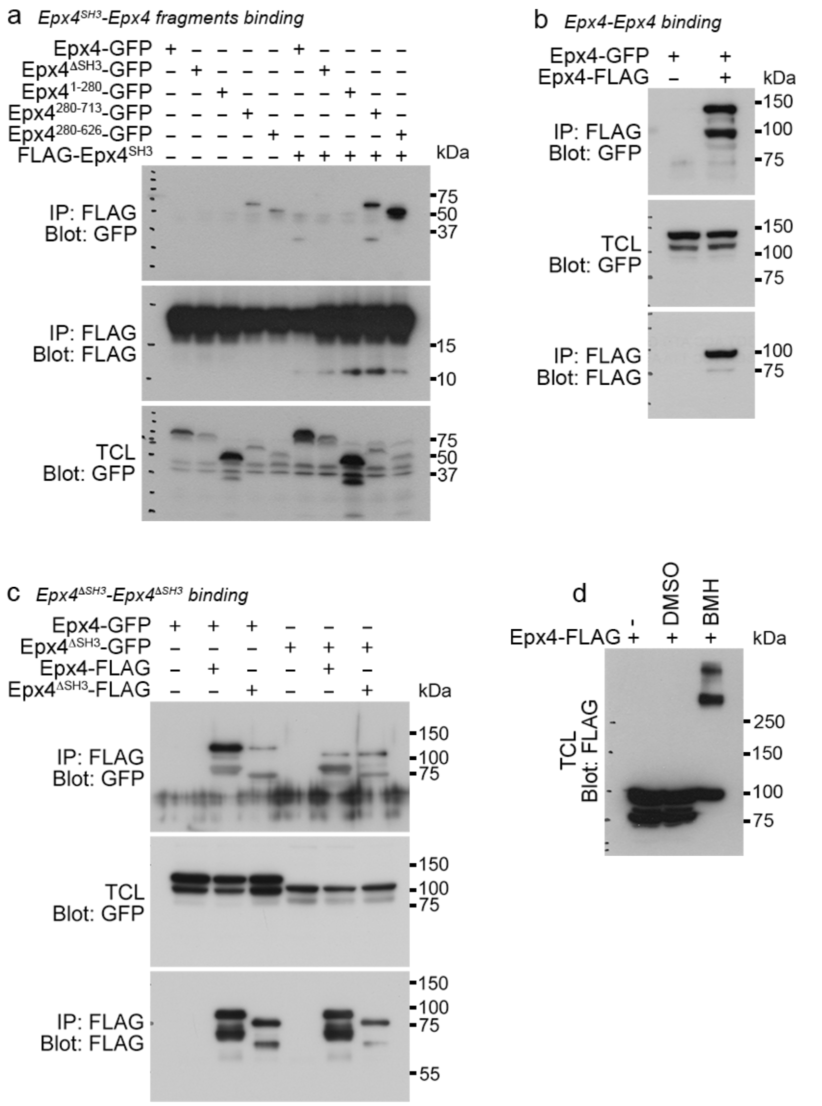

Uncropped western blots for the blots used in Figure 2

Supplementary S5

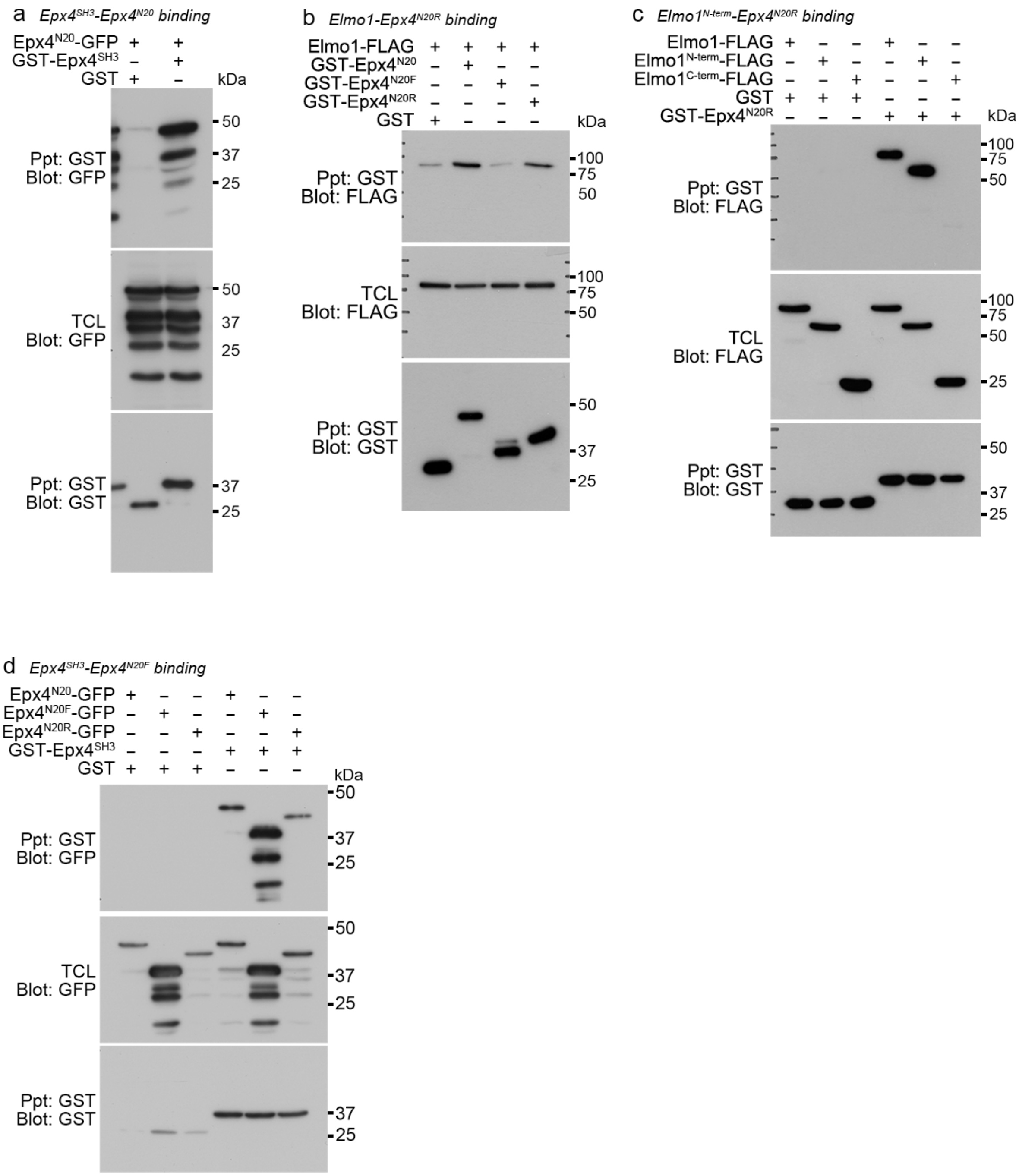

Uncropped western blots for the blots used in Figure 3

Supplementary S6

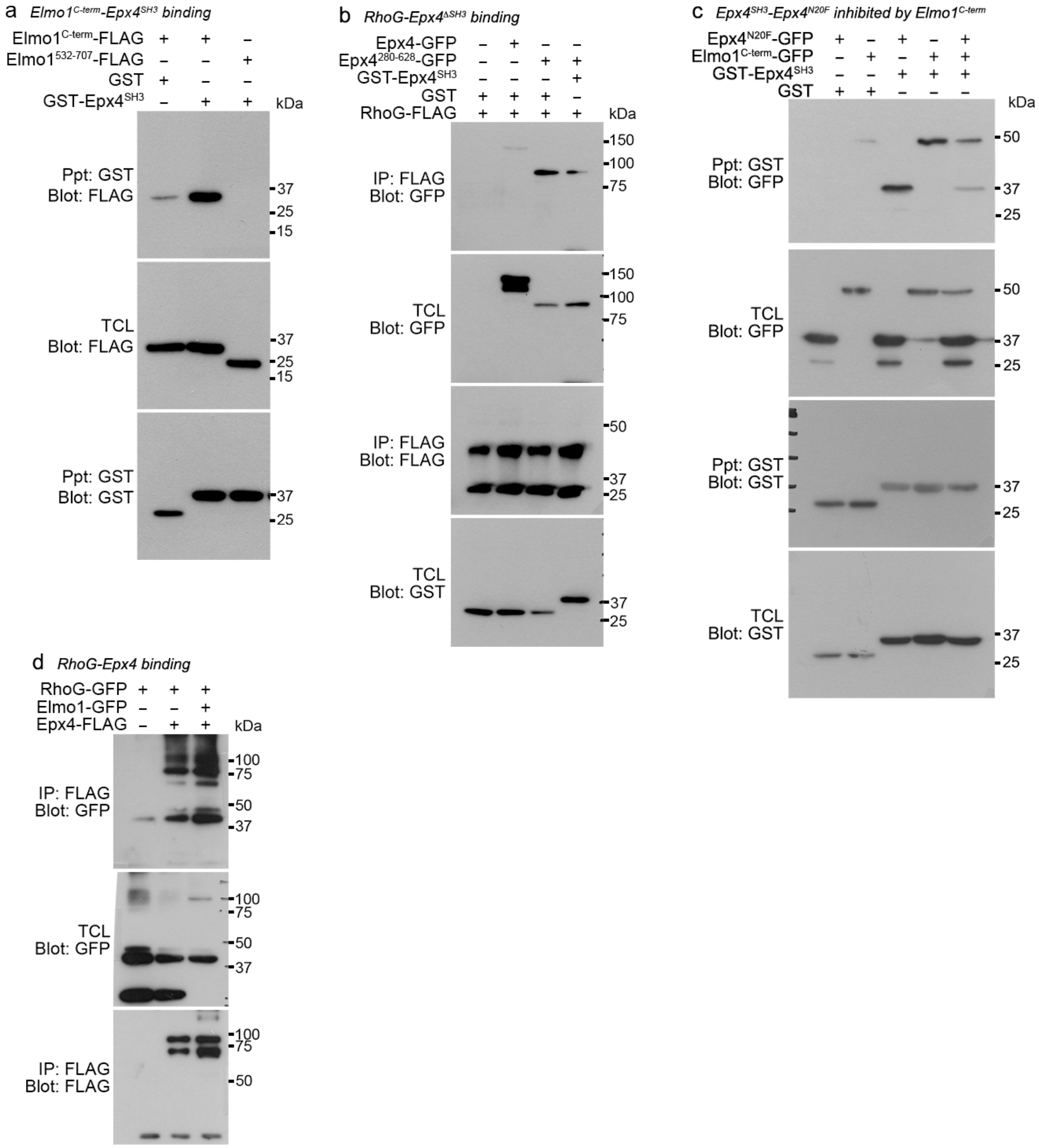

Uncropped western blots for the blots used in Figure 4

Supplementary S7

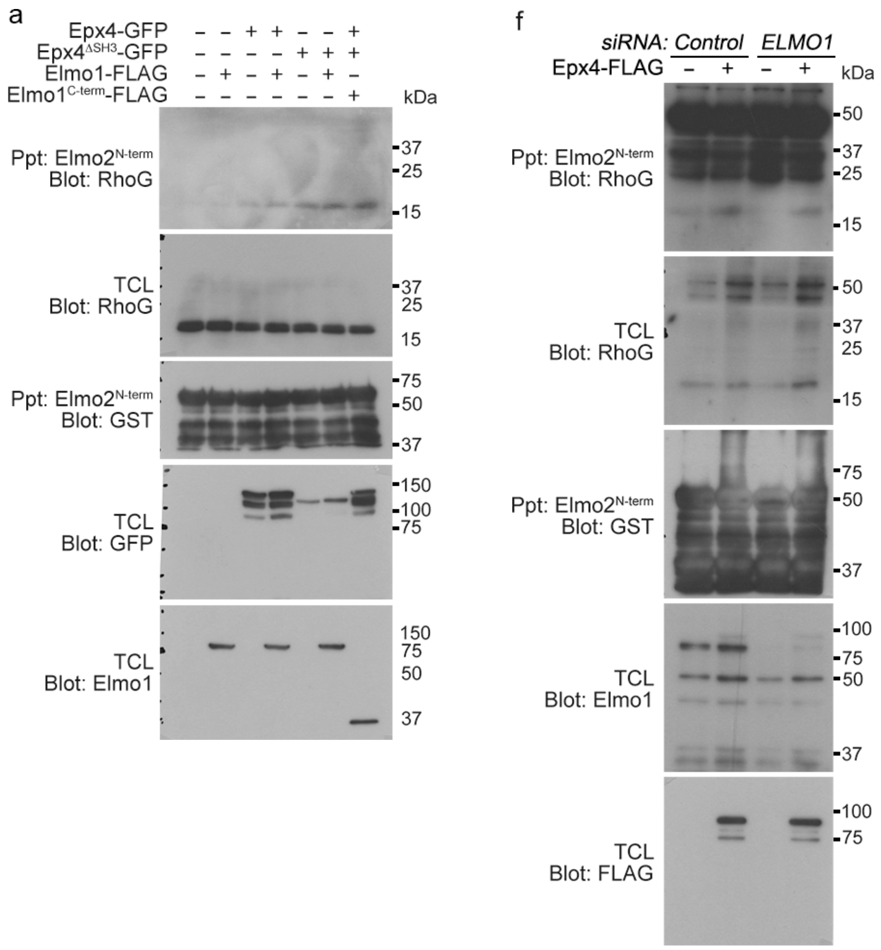

Uncropped western blots for the blots used in Figure 5

Supplementary S8

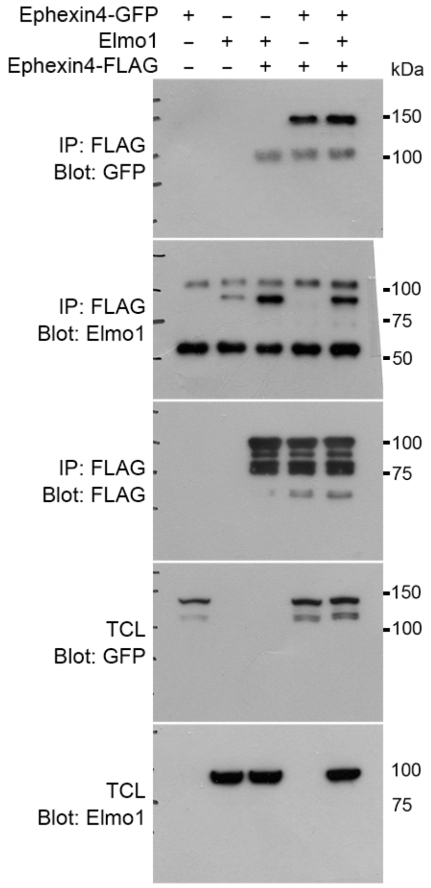

Uncropped western blots for the blots used in Supplementary S2
